# Lipid accumulation product and WHO risk chart-defined cardiovascular high-risk status in community adults: a cross-sectional analysis from the ChinaHEART program

**DOI:** 10.3389/fnut.2026.1865455

**Published:** 2026-07-23

**Authors:** Wenjuan Wu, Fangfang Hao, Shengming Huang, Jin Wang, Yijun Song, Haoran Wang

**Affiliations:** 1 Tianjin Medical University General Hospital, Tianjin, China; 2 State Key Laboratory of Experimental Hematology, Haihe Laboratory of Cell Ecosystem, Department of Intensive Care Medicine, National Clinical Research Center for Blood Diseases, Institute of Hematology & Blood Diseases Hospital, Chinese Academy of Medical Sciences & Peking Union Medical College, Tianjin, China; 3 Department of Neurology, The First Affiliated Hospital of Henan University of Science and Technology, Luoyang, China; 4 Department of Neurology, Hebei Petrochina Central Hospital, Langfang, China; 5 Cardiovascular Institute of Luohe, Luohe Medical College, Luohe Central Hospital, Luohe, China

**Keywords:** cardiovascular high-risk status, central adiposity, ChinaHEART, community screening, cross-sectional study, lipid accumulation product

## Abstract

**Background:**

Lipid accumulation product (LAP), derived from waist circumference and triglycerides, may reflect central lipid overaccumulation, but its relevance for a screening-defined cardiovascular disease (CVD) high-risk label is uncertain. We examined whether LAP could help flag community adults who may need more formal risk assessment rather than serve as a stand-alone prognostic tool.

**Methods:**

This cross-sectional analysis initially considered 6,860 adults aged 35–75 years from the ChinaHEART screening program in Luohe. After excluding participants with missing LAP or screening-defined CVD high-risk outcome data, 6,803 participants were included in the base analytic sample. LAP was analyzed continuously and dichotomized using a rounded cutoff of 42 derived from the Youden index in the LAP-only receiver operating characteristic (ROC) analysis. Logistic regression, restricted cubic splines, subgroup analyses, and ROC analyses were conducted under four stepwise models. Exploratory least absolute shrinkage and selection operator (LASSO), XGBoost, and TreeSHAP analyses were added as supplementary pattern-recognition analyses.

**Results:**

ROC analysis identified an optimal LAP cutoff of 42.12 in the LAP-only model; the rounded value of 42 was used for categorical analyses. LAP alone showed modest discrimination for the concurrent screening-defined CVD high-risk label (AUC 0.583, 95% CI 0.566–0.600), which increased to 0.628 (95% CI 0.613–0.643) after adding age and sex and to 0.632 (95% CI 0.619–0.649) in Model 3. Using the rounded cutoff of 42, 42.3% of participants were classified as high LAP. The prevalence of screening-defined CVD high-risk status was higher in the high-LAP group than in the low-LAP group (28 vs. 17%, *P* < 0.001).

**Conclusions:**

Higher LAP was associated with a greater likelihood of meeting a contemporaneous screening-defined CVD high-risk criterion. These findings support LAP as a pragmatic screening trigger for further formal assessment in community settings, not as evidence of causal risk stratification or prediction of hard cardiovascular events.

## 1 Introduction

Cardiovascular disease (CVD) remains a major cause of death and disability worldwide and imposes a substantial burden on health systems, especially in populations undergoing rapid aging and cardiometabolic transition (1–4). In community prevention programs, early identification of adults who merit more detailed evaluation is a central operational goal. Such programs do not simply seek to predict future hard outcomes; they often need practical markers that can help trigger more formal multivariable risk assessment during screening encounters.

Obesity, particularly central adiposity, is strongly linked to cardiometabolic dysfunction and adverse vascular health (3, 4). Conventional indices such as body mass index (BMI) and waist circumference (WC) are easy to obtain but incompletely reflect metabolically active visceral fat. Lipid accumulation product (LAP), calculated from WC and fasting triglycerides (TG), integrates an anthropometric component with a biochemical component and has been proposed as a surrogate marker of lipid overaccumulation and visceral adiposity (5, 6). Prior studies have associated LAP with insulin resistance, metabolic syndrome, fatty liver disease, hypertension, and incident or prevalent cardiovascular conditions (7–12).

Nonetheless, the interpretation of LAP requires caution when the outcome of interest is not an adjudicated cardiovascular event but a screening-defined high-risk classification. LAP itself is a composite measure, and the screening outcome used in community programs is also a composite construct derived from multiple clinical domains. Under this circumstance, the relevant clinical question is not whether LAP independently predicts future events in a causal sense, but whether it can help flag individuals who may warrant more complete downstream risk evaluation.

Using baseline data from the Luohe site of the ChinaHEART program, we therefore reframed the present analysis around screening utility rather than event prediction. Specifically, we examined the association between LAP and screening-defined CVD high-risk status, characterized the dose–response pattern with restricted cubic splines, explored subgroup consistency, and assessed the discriminatory performance of simple stepwise models for identifying the concurrent high-risk screening label.

## 2 Materials and methods

### 2.1 Study design and population

This was a cross-sectional analysis of baseline data from the ChinaHEART screening program in Luohe, Henan Province, China (13, 14). A total of 6,860 adults aged 35–75 years were initially available in the Luohe dataset. For the present analysis, participants were first required to have non-missing data for both LAP and screening-defined CVD high-risk status. Fifty-seven participants had missing LAP, whereas no participant had missing CVD high risk status; therefore, the base analytic sample comprised 6,803 participants. Descriptive summaries were generated using available data for each variable. Regression, spline, and ROC analyses used model-specific complete cases according to the variables required for each model. Because LDL-C was missing in 178 participants, the exploratory Model 4 was based on 6,625 participants, whereas Models 1–3 retained 6,803 participants.

All participants provided written informed consent. The study protocol was approved by the ethics committee of Fuwai Hospital and filed with the local ethics committee of Luohe Central Hospital. The present report was prepared in accordance with the Strengthening the Reporting of Observational Studies in Epidemiology (STROBE) framework for observational studies (15).

### 2.2 Data collection and measurements

Trained staff collected questionnaire, anthropometric, blood pressure, and laboratory data using standardized procedures within the ChinaHEART program. Demographic and socioeconomic variables included age, sex, marital status, education, farmer status, and annual household income category. Lifestyle variables included current smoking and alcohol use. Clinical variables used in the present manuscript included systolic blood pressure (SBP), fasting glucose, low-density lipoprotein cholesterol (LDL-C), and stroke history, alongside other descriptive characteristics summarized in Table 1.

**Table 1:** Baseline characteristics by LAP group (cutoff = 42).

Group	Variable	Overall	Low LAP (<42)	High LAP (≥42)	*P*-value
		*N* = 6,803	*N* = 3,922	*N* = 2,881	
Demographics/Socioeconomic	Age (years)	58 (51, 66)	58 (50, 67)	59 (52, 66)	0.034
	Male sex	2,570 (38%)	1,749 (45%)	821 (28%)	<0.001
	Married	6,041 (89%)	3,518 (90%)	2,523 (88%)	0.007
	Higher education	5,515 (81%)	3,287 (84%)	2,228 (77%)	<0.001
	Farmer	3,996 (59%)	2,242 (57%)	1,754 (61%)	0.002
	Last year income >50k	3,559 (52%)	1,878 (48%)	1,681 (58%)	<0.001
Lifestyle	Current smoking	1,362 (20%)	905 (23%)	457 (16%)	<0.001
	Alcohol use	372 (5.5%)	189 (4.8%)	183 (6.4%)	0.007
Anthropometry/Vital signs	Height (cm)	159 (154, 166)	160 (155, 167)	158 (153, 164)	<0.001
	Weight (kg)	64 (58, 72)	61 (55, 68)	68 (62, 76)	<0.001
	Waist circumference (cm)	86 (80, 92)	82 (77, 87)	92 (86, 98)	<0.001
	Body mass index (kg/m^2^)	25.1 (23.0, 27.3)	23.7 (22.0, 25.6)	27.0 (25.1, 29.2)	<0.001
	Systolic blood pressure (mmHg)	137 (126, 150)	134 (123, 147)	143 (131, 152)	<0.001
	Diastolic blood pressure (mmHg)	83 (77, 91)	82 (75, 88)	86 (79, 94)	<0.001
	Heart rate (bpm)	75 (70, 83)	75 (70, 82)	76 (71, 84)	<0.001
Laboratory measurements	Total cholesterol (mmol/L)	4.82 (4.12, 5.50)	4.64 (3.99, 5.23)	5.06 (4.35, 5.86)	<0.001
	HDL-C (mmol/L)	1.43 (1.23, 1.66)	1.47 (1.27, 1.72)	1.36 (1.17, 1.57)	<0.001
	Triglycerides (mmol/L)	1.51 (1.14, 2.03)	1.22 (0.98, 1.49)	2.10 (1.69, 2.83)	<0.001
	LDL-C (mmol/L)	2.63 (2.07, 3.15)	2.61 (2.09, 3.03)	2.65 (2.04, 3.34)	<0.001
	Fasting glucose (mmol/L)	5.40 (5.19, 6.00)	5.30 (5.10, 5.80)	5.70 (5.30, 6.30)	<0.001
Medical history	Stroke	191 (2.8%)	105 (2.7%)	86 (3.0%)	0.493
	Hypertension	1,563 (23%)	625 (16%)	938 (33%)	<0.001
	Diabetes mellitus	425 (6.2%)	159 (4.1%)	266 (9.2%)	<0.001
Medication use	Antihypertensive medication	906 (13%)	352 (9.0%)	554 (19%)	<0.001
	Antidiabetic medication	264 (3.9%)	103 (2.6%)	161 (5.6%)	<0.001
	Statin use	206 (3.0%)	79 (2.0%)	127 (4.4%)	<0.001
Outcome	CVD high-risk status	1,475 (22%)	663 (17%)	812 (28%)	<0.001

Continuous variables are presented as median (Q1, Q3); dichotomous variables as *n* (%). Base sample required non-missing LAP and CVD high risk status only. Mann–Whitney *U* test for continuous variables; Pearson's Chi-squared test for categorical variables.

Height, weight, and waist circumference were measured according to the screening protocol. Blood pressure was measured after seated rest using standardized equipment. Blood samples were obtained after fasting and were used to determine lipid and glucose parameters. In the present analysis, all associations were interpreted against the program-recorded screening outcome rather than incident cardiovascular events.

### 2.3 Exposure definition

LAP was calculated using sex-specific formulas: LAP = (WC [cm] − 65) × TG [mmol/L] for men and LAP = (WC [cm] − 58) × TG [mmol/L] for women (6). Higher LAP values indicate greater combined central lipid accumulation.

In the primary analyses, LAP was modeled in two ways: as a continuous variable per 1-unit increase and as a binary variable using a rounded, data-driven operational cutoff derived from the Youden index in the LAP-only ROC analysis. This cutoff was used solely for internal stratified analyses within the present dataset to facilitate descriptive and regression comparisons. It should therefore be interpreted as a sample-specific analytical threshold rather than as an externally generalizable screening threshold or clinical decision boundary.

### 2.4 Outcome definition

The outcome was screening-defined CVD high-risk status derived from the laboratory-based WHO CVD risk charts calibrated for China. In the screening workflow, the chart estimated each participant's 10-year risk of a major cardiovascular event using age, sex, smoking status, diabetes status, averaged systolic blood pressure, and total cholesterol. Participants with an estimated 10-year risk of ≥20% were classified as having high CVD risk. In the present manuscript, this outcome was interpreted as a concurrent screening classification rather than as incident cardiovascular events or adjudicated prognosis.

### 2.5 Covariates and model framework

Four stepwise logistic models were prespecified to examine the robustness of the LAP–outcome association while minimizing overadjustment and circularity. Model 1 included LAP only. Model 2 adjusted for age and sex, which are fundamental demographic determinants of cardiovascular risk and potential confounders of the association between LAP and screening-defined high-risk status. Model 3 additionally adjusted for marital status, education, farmer status, and annual income greater than 50,000 RMB to account for socioeconomic and lifestyle-context differences that may influence both adiposity-related phenotypes and screening-defined cardiovascular risk.

Model 4 additionally included current smoking, alcohol use, systolic blood pressure, fasting glucose, LDL-C, and stroke history and was treated as an exploratory extension rather than the primary inferential model. This distinction was intentional because both the exposure and the outcome integrate conventional cardiometabolic information: LAP itself incorporates waist circumference and triglycerides, whereas the laboratory-based WHO CVD risk classification is derived from established cardiovascular risk factors. Accordingly, extensive adjustment for traditional clinical factors may partially condition on information closely related to the screening outcome itself, thereby attenuating associations through overadjustment or circularity. For this reason, Model 3 was prespecified as the primary adjusted model, whereas Model 4 was interpreted as a conservative stress-test model.

### 2.6 Statistical analysis

Continuous variables are presented as medians with interquartile ranges (IQRs), and dichotomous variables as counts and percentages. Baseline characteristics were compared between LAP groups using Mann–Whitney *U* tests for continuous variables and Pearson chi-squared tests for categorical variables.

Logistic regression was used to estimate odds ratios (ORs) and 95% confidence intervals (CIs) for screening-defined CVD high-risk status according to continuous LAP and binary LAP. For the main regression table, standard logistic regression was used, with bias-reduced logistic regression available automatically when model instability occurred.

Restricted cubic spline models were fitted for continuous LAP under the same four stepwise model structures, using four knots placed at the Harrell-recommended percentiles (5th, 35th, 65th, and 95th percentiles) of the model-specific LAP distribution. The reference LAP value was the sample median. Overall and non-linearity *P* values were obtained from model-based Wald tests.

Exploratory subgroup analyses displayed stratum-specific unadjusted ORs for LAP across sex, age group, current smoking, alcohol use, hypertension, and diabetes mellitus strata. Additional exploratory interaction panels visualized predicted probabilities across LAP according to quartiles of SBP, diastolic blood pressure (DBP), TG, total cholesterol, high-density lipoprotein cholesterol (HDL-C), and LDL-C. These subgroup and interaction displays were intended to illustrate screening patterns rather than to support definitive effect-modification claims.

ROC curves were used to assess discrimination for the concurrent screening-defined high-risk label under four stepwise models. Bootstrap resampling (1,000 iterations) was used to obtain 95% CIs for AUC estimates and the Model 1 LAP cutoff. Because model-specific complete-case samples differed across models, pairwise ROC comparisons used unpaired DeLong tests. As a supplementary sensitivity analysis responding to potential concerns about the overlap between Model 4 covariates and the WHO risk chart components, we evaluated the incremental discriminative value of adding LAP to a baseline logistic model containing only the Model 4 covariates without LAP. The continuous net reclassification improvement (NRI), integrated discrimination improvement (IDI), and decision curve analysis (DCA) were computed to assess whether LAP provided incremental classification benefit beyond the conventional risk factors already included in Model 4. All analyses were conducted in R 4.5.2 (R Foundation for Statistical Computing, Vienna, Austria), and two-sided *P* values <0.05 were considered statistically significant.

As a supplementary descriptive analysis, diagnostic performance metrics—including sensitivity, specificity, positive predictive value, and negative predictive value—were computed at the LAP cutoff derived from the Youden index to further characterize its screening properties.

As an exploratory supplementary analysis, we also implemented a conservative machine-learning workflow to assess whether LAP remained salient when direct or near-direct components of the composite screening label were intentionally excluded from the candidate pool. The candidate variables were restricted to LAP, marital status, education, farmer status, last-year income greater than 50k, alcohol use, BMI, heart rate, fasting glucose, diabetes mellitus history, and antidiabetic medication use. Using the same base sample requirement of non-missing LAP and CVD high risk status, continuous predictors were median-imputed and categorical predictors were coded with an explicit Missing level. Ten-fold cross-validated least absolute shrinkage and selection operator logistic regression using AUC was applied for feature screening; the selected features were then entered into XGBoost, with discrimination evaluated in a 70/30 train–test split. Exact TreeSHAP values derived from the fitted XGBoost model were used to rank feature importance. This workflow was prespecified as exploratory and hypothesis-generating, and was not intended to replace the primary regression-based screening analyses or to establish a deployable prediction model.

## 3 Results

### 3.1 Screening discrimination and cutoff determination

**Figure 1:**
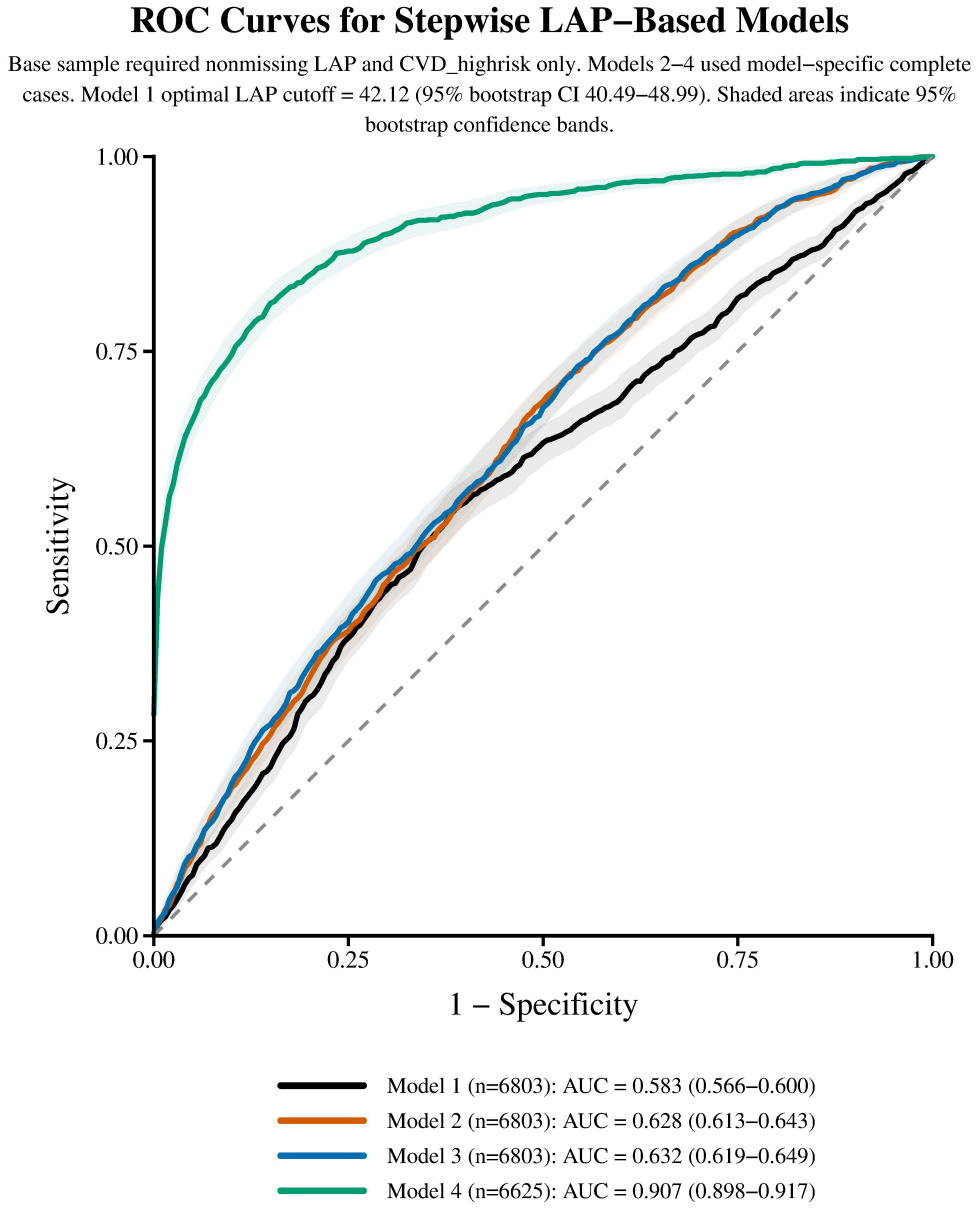
ROC curves for stepwise LAP-based models in identifying screening-defined CVD high-risk status. Model 1 included LAP only; Model 2 additionally included age and sex; Model 3 additionally included marital status, education, farmer status, and income; and Model 4 additionally included current smoking, alcohol use, systolic blood pressure, fasting glucose, LDL-C, and stroke history. The Model 1 sample-derived LAP cutoff was 42.12 and was rounded to 42 for categorical analyses.

In ROC analyses, the LAP-only model yielded a data-driven Youden index threshold of 42.12 (95% CI 40.49–48.99). For analytic convenience, this value was rounded to 42 and used only for categorical analyses within the current dataset, rather than being treated as an externally applicable screening threshold. LAP alone showed modest discrimination for the concurrent screening-defined CVD high-risk label, with an AUC of 0.583 (95% CI 0.566–0.600). At the rounded cutoff of 42, LAP demonstrated a sensitivity of 0.551 (95% CI 0.524–0.573), specificity of 0.612 (95% CI 0.599–0.624), PPV of 0.282 (95% CI 0.265–0.298), and NPV of 0.831 (95% CI 0.819–0.842; Supplement Table S1). Adding age and sex increased the AUC to 0.628 (95% CI 0.613–0.643), and further inclusion of demographic and socioeconomic covariates in Model 3 yielded an AUC of 0.632 (95% CI 0.619–0.649). The exploratory Model 4 reached an AUC of 0.907 (95% CI 0.898–0.917), but this result was interpreted cautiously because the model included several conventional clinical factors closely aligned with the operational screening label (Figure 1). The incremental value analysis confirmed this interpretation: adding LAP to the baseline Model 4 covariates without LAP produced a negligible change in AUC (ΔAUC = +0.0004; DeLong *P* = 0.122), a near-zero continuous NRI (−0.004; *P* = 0.901), and a non-significant IDI (+0.0005; *P* = 0.241). Decision curve analysis likewise showed virtually overlapping net benefit curves across a clinically relevant range of threshold probabilities (Supplementary Figure S1). These findings indicate that in the presence of conventional risk factors overlapping with the WHO risk chart components, LAP does not provide meaningful incremental discrimination or reclassification.

### 3.2 Baseline characteristics

Using this ROC-derived cut-off, 3,922 participants (57.7%) were classified as having low LAP and 2,881 (42.3%) as having high LAP. Compared with the low-LAP group, individuals with high LAP tended to be slightly older and more often female, and they showed a clear clustering of adverse cardiometabolic features, including higher BMI and waist circumference, higher systolic and diastolic blood pressure, and a more unfavorable lipid profile characterized by higher triglycerides and total cholesterol together with lower HDL-C. Fasting glucose and the prevalence of hypertension and diabetes mellitus were also higher in the high-LAP group. In addition, the prevalence of CVD high-risk status was markedly higher among participants with high LAP than among those with low LAP (28 vs. 17%, *P* < 0.001). These findings suggest that higher LAP is accompanied by a less favorable overall cardiometabolic profile and a greater burden of screening-defined cardiovascular high-risk status (Table 1).

Specifically, participants with high LAP had higher BMI, waist circumference, SBP and DBP, total cholesterol, triglycerides, LDL-C, fasting glucose, and higher prevalences of hypertension, diabetes mellitus, and related medication use, whereas HDL-C was lower. These differences indicate that LAP captured clustering of central adiposity and metabolic disturbance in this community screening population, which is consistent with its intended role as a simple trigger for more formal cardiovascular risk assessment.

### 3.3 Association of LAP with screening-defined CVD high-risk status

In logistic regression analyses, LAP was positively associated with screening-defined CVD high-risk status across the stepwise models (Table 2). For continuous LAP, the OR per 1-unit increase was 1.007 (95% CI 1.006–1.009) in Model 1, 1.009 (95% CI 1.007–1.010) in Model 2, 1.009 (95% CI 1.007–1.011) in Model 3, and 1.003 (95% CI 1.001–1.006) in Model 4.

**Table 2:** Association between lipid accumulation product (LAP) and CVD high-risk in logistic regression models.

	Model 1		Model 2		Model 3		Model 4	
Variable	OR (95% CI)	*P* value	OR (95% CI)	*P* value	OR (95% CI)	*P* value	OR (95% CI)	*P* value
LAP (per 1-unit increase)	1.007 (1.006, 1.009)	<0.001	1.009 (1.007, 1.010)	<0.001	1.009 (1.007, 1.011)	<0.001	1.003 (1.001, 1.006)	0.016
High LAP (≥42) vs. Low LAP (<42)	1.929 (1.717, 2.167)	<0.001	2.083 (1.847, 2.351)	<0.001	2.101 (1.861, 2.372)	<0.001	1.327 (1.121, 1.570)	0.001

Model 1: crude (LAP only). Model 2: adjusted for age and sex. Model 3: additionally adjusted for Married, education, Farmer, and Last year income greater than 50,000RMB. Model 4: additionally adjusted for Current smoking, alcohol use, systolic blood pressure, glucose, low-density lipoprotein cholesterol, and history of stroke. Binary LAP group used cutoff = 42 (High: LAP≥42; Low: LAP <42). Base sample required non-missing LAP and CVD high risk status only; each model then used model-specific complete-case analysis.

When LAP was categorized at 42, high LAP showed stronger between-group contrast. Compared with low LAP, high LAP was associated with higher odds of the screening-defined high-risk label in Model 1 (OR 1.929, 95% CI 1.717–2.167), Model 2 (OR 2.083, 95% CI 1.847–2.351), Model 3 (OR 2.101, 95% CI 1.861–2.372), and Model 4 (OR 1.327, 95% CI 1.121–1.570).

The attenuation from Model 3 to Model 4 suggests that part of the observed association is shared with conventional clinical information already closely related to the screening-defined high-risk label. Even so, the persistence of the association in Model 4 supports the view that LAP can still function as a pragmatic screening enrichment marker rather than a formal stand-alone stratification tool.

### 3.4 Restricted cubic spline analysis

**Figure 2:**
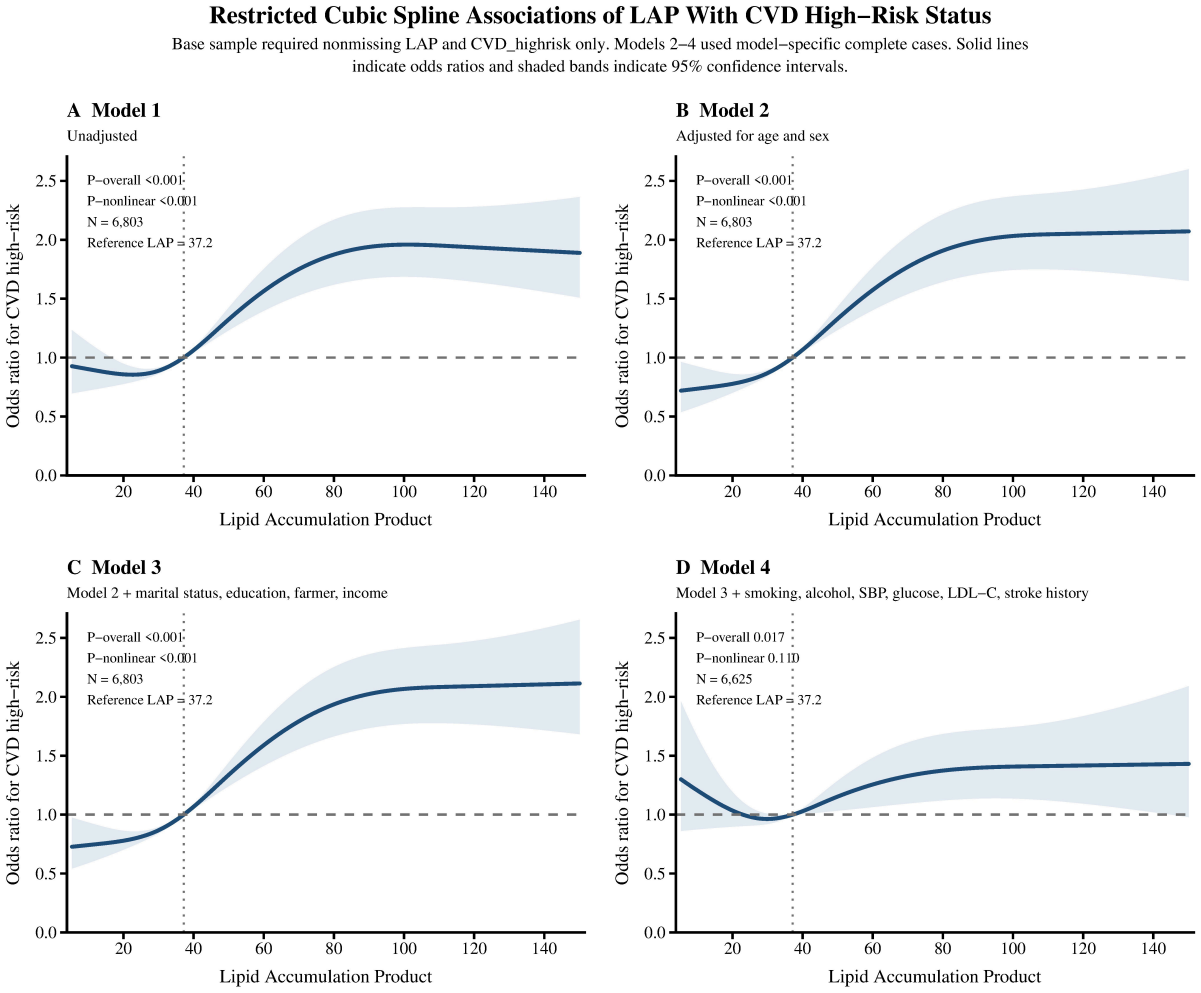
Restricted cubic spline associations between LAP and screening-defined CVD high-risk status across four stepwise models: **(A)** Model 1; **(B)** Model 2; **(C)** Model 3; and **(D)** Model 4. Models 1–3 showed significant overall association and non-linearity, whereas Model 4 retained a weaker overall association with attenuated non-linearity.

Restricted cubic spline analyses provided additional detail on the dose–response pattern (Figure 2). In Models 1–3, the overall association and the non-linearity test were both statistically significant (all *P*-overall <0.001; all *P*-non-linear <0.001), indicating a non-uniform increase in the odds of the screening-defined high-risk label across the LAP range.

After additional exploratory adjustment in Model 4, the overall association remained statistically significant (*P*-overall = 0.017), whereas evidence of non-linearity was attenuated (*P*-non-linear = 0.110). This pattern suggests that a threshold-like shape was evident before the more conventional clinical factors in Model 4 were introduced, but that some of this apparent curvature was shared with those accompanying risk characteristics.

### 3.5 Exploratory subgroup, interaction, and machine-learning analyses

**Figure 3:**
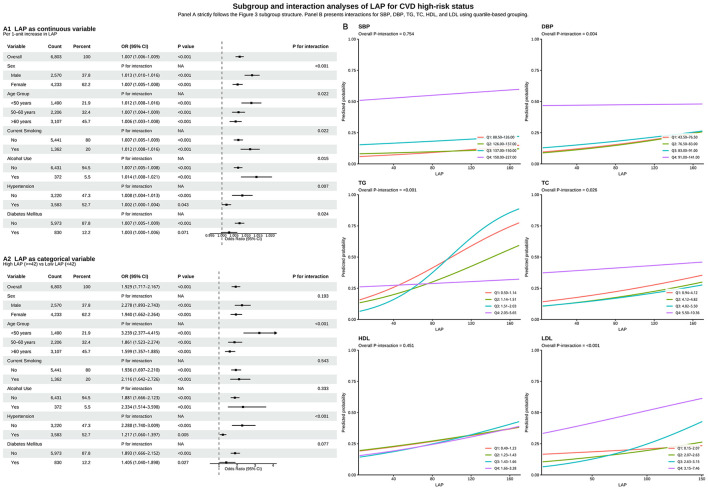
Exploratory subgroup and interaction analyses of LAP for screening-defined CVD high-risk status. Panel **A1** shows unadjusted subgroup ORs for continuous LAP; Panel **A2** shows unadjusted subgroup ORs for high vs. low LAP using the cutoff of 42; Panel **B** shows exploratory interaction curves across quartiles of conventional cardiometabolic factors.

Exploratory subgroup analyses are shown in Figure 3. The direction of association between LAP and the screening-defined high-risk label was generally consistent across strata of sex, age group, current smoking, alcohol use, hypertension, and diabetes mellitus. Relative associations appeared stronger in younger participants, although these analyses were descriptive and should not be over-interpreted.

In the interaction panels, higher predicted probabilities of the screening-defined high-risk label were observed across increasingly adverse quartiles of SBP, DBP, TG, total cholesterol, HDL-C, and LDL-C. These findings support the interpretation that LAP tracks with broader metabolic burden in the screening setting, but they do not establish mechanistic interaction or incremental prognostic synergy.

**Figure 4:**
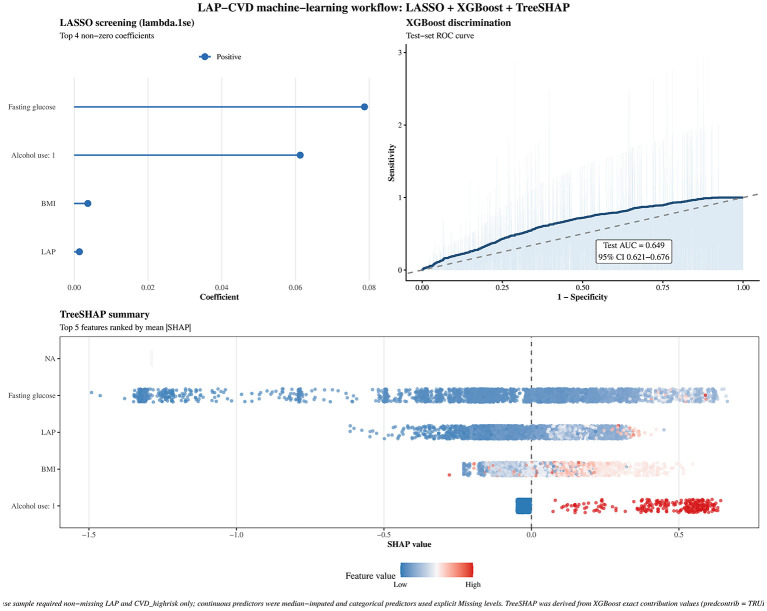
Exploratory LASSO + XGBoost + TreeSHAP analysis under a conservative non-overlap candidate pool. Candidate variables deliberately excluded direct or near-direct components of the composite screening outcome to reduce circular performance inflation. LASSO was used for feature screening, XGBoost for exploratory discrimination, and exact TreeSHAP values for feature ranking. Findings should be interpreted as exploratory pattern recognition rather than formal risk prediction.

Figure 4 summarizes the exploratory machine-learning workflow. Under the conservative non-overlap candidate pool, LASSO retained four non-zero features at lambda.1se: LAP, BMI, alcohol use, and fasting glucose. XGBoost built from these screened features yielded a test-set AUC of 0.649 (95% CI 0.621–0.676), and TreeSHAP ranked alcohol use, BMI, LAP, and fasting glucose as the most influential features. These exploratory findings support the persistence of LAP as a salient screening-related feature, but not as a basis for claiming a validated clinical prediction model.

## 4 Discussion

### 4.1 Principal findings

In this community-based cross-sectional analysis of ChinaHEART screening data, higher LAP was consistently associated with a greater likelihood of meeting the screening-defined CVD high-risk criterion. High-LAP participants had a distinctly less favorable cardiometabolic profile and a higher prevalence of the screening-defined CVD high-risk label at the descriptive level (28 vs. 17%), both continuous and binary LAP were positively associated with the screening label across the stepwise models, and the dose–response pattern showed threshold-sensitive non-linearity before extensive clinical conditioning. These findings support LAP as an accessible signal of accumulated metabolic burden in community screening settings.

The main interpretive point, however, is not that LAP predicts future events or establishes causal risk. Rather, LAP appears to mark individuals who are more likely to already satisfy a composite screening-defined high-risk classification and who may therefore deserve more formal downstream evaluation.

### 4.2 Comparison with previous studies and possible explanations

Our results are broadly aligned with prior studies linking LAP to metabolic syndrome, fatty liver disease, hypertension, and cardiovascular outcomes (7–12, 16, 17). LAP combines WC and TG, thereby capturing both central adiposity and a dyslipidemic component. This is biologically plausible as a marker of metabolic dysregulation because visceral fat accumulation is associated with inflammation, altered adipokine signaling, hepatic free fatty acid flux, insulin resistance, and a more atherogenic metabolic milieu (3, 4, 18–21); moreover, emerging evidence highlights the roles of mitochondrial dysfunction, subclinical vascular injury, exposome-related factors, and psychosocial stress in shaping cardiovascular risk beyond traditional metabolic pathways (22–25).

At the same time, the present study differs from event-based cohort analyses. The current outcome is a screening-defined composite label, not incident myocardial infarction, stroke, or cardiovascular death. Accordingly, the observed association likely reflects overlap in broader cardiometabolic burden rather than a discrete causal pathway from LAP to hard cardiovascular events. The attenuation seen after additional exploratory adjustment is fully consistent with this interpretation.

### 4.3 Interpretation boundary: screening trigger, not stand-alone stratification

The distinction between a screening trigger and a prognostic model is critical here. First, LAP is itself a composite index. Second, the outcome is also composite and operational, designed for screening rather than for adjudicating future clinical events. Third, the excellent discrimination of Model 4 (AUC = 0.907) is driven primarily by variables that are integral components of the WHO risk chart itself (age, sex, systolic blood pressure, fasting glucose, LDL-C, and smoking status) rather than by LAP; the supplementary incremental value analysis confirmed that LAP contributed negligible additional discrimination beyond these established risk factors (ΔAUC = +0.0004). For these reasons, the present findings should not be described as evidence that LAP independently predicts hard cardiovascular endpoints, nor should they be used to justify stand-alone risk stratification.

A more defensible interpretation is that LAP may help enrich screening encounters. Rather than serving as a stand-alone predictor or replacing established risk assessment tools, LAP may function as a pragmatic extension or complement to the WHO risk chart in community settings where complete laboratory panels are not immediately available. In settings where rapid triage is needed and only simple measurements are immediately available, an elevated LAP may serve as a cue to proceed with more complete risk assessment, reinforce counseling, or prioritize additional blood pressure and laboratory review.

### 4.4 Clinical and public health implications

From an implementation perspective, LAP has practical advantages. It can be calculated from measurements that are already routine in community programs, imposes little additional cost, and summarizes central adiposity and TG in a single number. The sample-derived cutoff around 42 should be viewed only as an internal analytical threshold for the present study, primarily used to structure categorical comparisons within this dataset. It should not be interpreted as an externally applicable screening threshold or clinical decision boundary without independent validation in other populations and screening settings.

Nevertheless, the cutoff should not be exported uncritically into routine practice. The modest sensitivity (0.551) and low PPV (0.282) at the rounded cutoff of 42 confirm that LAP alone is insufficient as a stand-alone diagnostic instrument; its primary screening value lies in its high NPV (0.831), which may support its use as a rule-out tool in community triage settings. Because it was derived and evaluated in the same cross-sectional population, external validation is required. If LAP is used in practice at all, it should be positioned as an adjunctive trigger for fuller assessment rather than a substitute for comprehensive multivariable risk evaluation.

### 4.5 Strengths and limitations

This study has several strengths. It was based on a relatively large community screening sample with standardized measurements. The analytic framework is transparent, uses stepwise models that separate demographic and socioeconomic adjustment from exploratory clinical conditioning, and triangulates the association using logistic regression, restricted cubic splines, subgroup displays, and ROC analyses.

The study also has important limitations. First, the cross-sectional design precludes causal inference and cannot address future event prediction. Second, both the exposure and the outcome are composite constructs, which creates interpretive overlap and makes strong mechanistic conclusions inappropriate. Third, the high AUC of Model 4 (0.907) should not be interpreted as evidence of LAP's predictive ability, as confirmed by the supplementary incremental value analysis showing negligible ΔAUC, NRI, and IDI; this high AUC is attributable to the WHO risk chart constituent variables rather than to LAP. Fourth, the subgroup and interaction displays were exploratory and largely unadjusted. Finally, the LAP cutoff was data-driven and derived within the same cross-sectional dataset in which it was subsequently evaluated, introducing potential optimism and limiting transportability. Fifth, complete-case analyses were used for all regression and ROC models; LDL-C was missing in 178 participants (2.6% of the base sample), and although this proportion is relatively small, complete-case analysis may introduce selection bias if missingness is not completely at random. Sensitivity analyses using multiple imputation were not performed, which may limit the robustness of Model 4 estimates. This threshold should therefore be regarded as an internal analytical cutoff for the present study rather than as a broadly applicable external threshold, and it requires independent external validation before any wider use.

## 5 Conclusions

In this community-based ChinaHEART screening population, higher LAP was associated with a greater likelihood of meeting the concurrent screening-defined CVD high-risk criterion. The association was robust in the primary adjusted model, remained statistically significant after exploratory clinical adjustment, and showed evidence of non-linearity before extensive conditioning.

These findings support LAP as a simple screening trigger that may help identify individuals who warrant more formal CVD risk assessment in community settings. They do not support causal claims, prediction of hard cardiovascular endpoints, or use of LAP as a stand-alone risk stratification tool. Prospective studies and external validation are needed before translating the present cutoff or model results into broader clinical practice.

## Data Availability

The raw data supporting the conclusions of this article will be made available by the authors, without undue reservation.

## Supplementary material

Supplementary Table S1 Diagnostic performance of LAP (cutoff = 42) for identifying screening-defined CVD high-risk status. Sensitivity, specificity, positive predictive value, and negative predictive value are reported with 95% bootstrap confidence intervals.

Supplementary Figure S1 Incremental value analysis of adding LAP to Model 4 covariates. Panel A shows ROC curves for Model 4 with and without LAP; Panel B presents decision curve analysis over a clinically relevant range of threshold probabilities. NRI (continuous), IDI, and ΔAUC with DeLong test *P* values are reported.
