# Supplementary material for: Lipid accumulation product and WHO risk chart-defined cardiovascular high-risk status in community adults: a cross-sectional analysis from the ChinaHEART program
Source: Front Nutr. 2026 Jul 23;13:1865455. doi: 10.3389/fnut.2026.1865455 (PMC13441729; doi:10.3389/fnut.2026.1865455)
Supplement: Supplementary file 1 [file Table_1.docx]

***Supplementary Table S1.*** *Diagnostic performance of LAP (cutoff = 42) for identifying screening-defined CVD high-risk status*

| **Metric** | **Value** | **95% CI** |
| --- | --- | --- |
| Sample size (N) | 6803 | — |
| CVD high-risk prevalence | 21.7% | — |
| Sensitivity | 0.551 | 0.524 – 0.573 |
| Specificity | 0.612 | 0.599 – 0.624 |
| Positive predictive value (PPV) | 0.282 | 0.265 – 0.298 |
| Negative predictive value (NPV) | 0.831 | 0.819 – 0.842 |
| LAP cutoff = 42, derived from the Youden index in the LAP-only ROC analysis and rounded for categorical use. 95% CIs obtained from 2,000 bootstrap resamples. PPV and NPV depend on the sample prevalence of CVD high-risk status (21.7%) and should not be extrapolated to populations with different risk profiles. Abbreviations: LAP, lipid accumulation product; CVD, cardiovascular disease; CI, confidence interval; PPV, positive predictive value; NPV, negative predictive value. | | |
